# Evaluation of surrogacy in the multi-trial setting based on information theory: an extension to ordinal outcomes

**DOI:** 10.1080/10543406.2019.1696357

**Published:** 2019-12-30

**Authors:** Hannah Ensor, Christopher J. Weir

**Affiliations:** Edinburgh Clinical Trials Unit, Usher Institute, University of Edinburgh, Edinburgh, UK

**Keywords:** Clinical trials, information theory, surrogacy evaluation

## Abstract

Summary: In clinical trials, surrogate outcomes are early measures of treatment effect that are used to predict treatment effect on a later primary outcome of interest: the primary outcome therefore does not need to be observed and trials can be shortened. Evaluating surrogates is a complex area as a given treatment can act through multiple pathways, some of which may circumvent the surrogate. One of the best established and practically sound approaches to surrogacy evaluation is based on information theory. We have extended this approach to the case of ordinal outcomes, which are used as primary outcomes in many medical areas. This extension provides researchers with the means of evaluating surrogates in this setting, which expands the usefulness of the information theory approach while also demonstrating its versatility.

## 1. Introduction

It is legitimate to use a surrogate in place of the true or primary outcome of interest in a clinical trial if it can be established that it informs on the treatment effect on the true outcome. In so doing, a clinical trial can be conducted with a smaller sample size and efficacious treatments can be made available to patients in a more timely fashion. However, the confidence placed in a “legitimate” surrogate can only be as strong as the means of establishing its validity. Baker and Kramer (2003) stated that where treatments work through multiple pathways (as is often the case) surrogacy assessment is difficult. Many different approaches for the evaluation of surrogates have been suggested (Alonso and Molenberghs 2007; Frangakis and Rubin 2004; Molenberghs et al. 2008; Robins and Greenland 1992). For a systematic review of methods see Ensor et al. (2016). These tend to examine whether surrogates are informative at both the individual patient level and the clinical trial level.

The work presented here aims to extend multi-trial information theory-based surrogate evaluation to the case of ordinal outcomes. In so doing we allow researchers to evaluate surrogates in areas where ordinal outcomes are used, for instance in stroke where the Oxford Handicap Scale (Bamford et al. 1989) is often measured.

Various existing surrogate evaluation approaches could be extended to the case of ordinal outcomes, including the direct and indirect effects, principal stratification and information theory approaches (Alonso and Molenberghs 2007; Frangakis and Rubin 2004; Robins and Greenland 1992). Aside from quantitatively evaluating the potential surrogate, we would wish any such approach to have four main properties. An approach should be: (a) practically viable; (b) able to inform on the causal nature of relationships between the surrogate and true outcome; (c) able to identify the surrogate paradox. The surrogate paradox occurs when there are positive treatment effects on the surrogate, and a positive relationship between the surrogate and true outcome, but a negative treatment effect on the true outcome; and (d) inform on the surrogate’s transportability or predictive ability, a fundamental requirement of surrogacy whereby surrogates evaluated in one trial would be able to inform on the treatment effect on the true outcome in a new trial.

Pragmatic multi-trial approaches, including meta-analytical (Buyse et al. 2000) and information theory (Alonso and Molenberghs 2007) are well-established methods that fulfil to a good standard all the above criteria. Therefore, we consider the multi-trial approaches to be the most appropriate for extension to ordinal outcomes. These approaches assess surrogacy at two levels: the individual patient and trial levels. In simple terms, correlation is an insufficient measure of surrogacy because it ignores treatment mechanisms of action and can lead to the surrogate paradox. In multi-trial approaches, the individual patient measure of surrogacy is essentially a correlation but treatment allocation is taken into account. At the trial level, multi-trial approaches provide a measure of the predictive ability of the surrogate to determine whether a surrogate could inform on the likely treatment effect on the primary outcome in a new trial, i.e. its transportability. This satisfies one of the primary aims of a valid surrogate. Combined these measures provide a methodologically sound and practically useful assessment of surrogacy that goes beyond a simple measurement of correlation.

The multi-trial information theory approach has fewer computational and interpretational issues compared to earlier multi-trial approaches and provides consistent interpretation across settings (for example, ordinal or continuous outcomes) (Alonso and Molenberghs 2007). Given these strong methodological and practical advantages, we select the multi-trial information theory approach here for extension to the case of ordinal outcomes.

Most methodology developed for ordinal outcomes has been in early surrogate evaluation measures such as single trial studies (Molenberghs et al. 2001) or the multi-trial (meta-analytical) approach (Burzykowski et al. 2003; Molenberghs et al. 2002; Renard et al. 2002); none have been evaluated via simulation. Under the meta-analytical approach Renard et al. (2002) briefly outline a latent variable approach; Burzykowski et al. (2003) present methodology for an ordinal surrogate and time to event true outcome; and Alonso et al. (2002) investigate the setting where one of the surrogate or true outcome is ordinal and the other continuous. In contrast, our work provides a fully developed methodological extension to the ordinal case in the multi-trial setting, building on the established strengths of the information theory approach to surrogacy evaluation. This has been evaluated by an extensive simulation study incorporating many settings not investigated previously, including weak strengths of surrogacy; discordant strengths of surrogacy at trial and individual levels; ceiling effects for categorical outcomes; as well as an investigation of the impact of non-proportional odds.

In Section 2 we outline the information theory approach and how this can be extended to the case of ordinal outcomes. We cover the “binary-ordinal” setting where the surrogate is binary and the true outcome ordinal; the theory developed could also be applied to the ordinal-ordinal setting with some minor modifications. Section 3 presents a simulation study to evaluate the properties of the ordinal extension. Section 4 illustrates the method using a case study from the stroke clinical trial CLOTS3 (Dennis et al. 2015) and Section 5 discusses our methodology extension in the broader context.

## 2. Methods

In what follows, the surrogate is denoted S, treatment is Z and the true outcome is T. There are *i* = 1,2, …,*N* trials, and *j* = 1,2, … .,$n_{i}$ patients per trial. $N_{T}=\sum_{i}n_{i}$ is the total number of patients in all trials. The ordinal true outcome has Wordered categories.

### 2.1. The information theory approach

Alonso and Molenberghs (2007) proposed an information theory surrogate evaluation measure based on the concepts of entropy and information theory by Shannon (1948). Information theory uses the central concept of entropy to measure the “information, choice and uncertainty” in a random variable. In the discrete case, entropy can be represented as $H\left(Y\right)=-\sum_{b\;=\;1}^{m_{y}}p_{b}log\left(p_{b}\right)$, where Y is a discrete random variable with values $k_{1},k_{2},\ldots .,k_{m_{y}}$ and probabilities $p_{1},p_{2},\ldots .,p_{m_{y}}$ respectively. Conditional$,H\left(Y|X\right)$, and joint entropy, $H\left(Y,X\right)$, can be straightforwardly defined. And differential entropy measures information in the continuous case, $h_{d}\left(Y\right)=-\int_{-\infty }^{\infty }f_{y}\left(y\right)\log\left\{f_{y}\left(y\right)\right\}dy$.

A concept of fundamental importance is the mutual information. This is defined as $I\left(X,Y\right)=H\left(Y\right)-H(Y|X)$ and is interpreted as the amount of uncertainty in Y removed if X is known. Another useful concept for comparing random variables is the entropy power, obtained by maximising the entropy of a continuous random variable, defined as $EP\left(Y\right)=\;\frac{1}{\left(2\pi e\right)}e^{2h\left(Y\right)}$. See Shannon (1948) for a full list of the properties of entropy and the mutual information.

These concepts are useful in surrogate evaluation as, at the individual level, we are interested in the amount of information on T (or ‘treatment effects on T’ at the trial level) covered by our knowledge of S (or ‘treatment effects on S’ at the trial level).

#### 2.1.1. Individual level: information theory approach

At the individual level, Alonso and Molenberghs (2007) proposed an information theory surrogate evaluation measure:

(1) $$R_{h}^{2}=\frac{EP\left(T\right)-EP\left(T|S\right)}{EP\left(T\right)}$$

where $EP\left(T\right)$ is the entropy power of T and $EP\left(T|S\right)$ is the entropy power of T given S. This can be interpreted as the amount of uncertainty in the true outcome T removed when S is known. $R_{h}^{2}$ has useful properties: it is linked to the mutual information through $R_{h}^{2}=1-e^{2I\left(S,T\right)}$; $R_{h}^{2}$ is invariant by bijective transformations of S and T; and $R_{h}^{2}=0$ if and only if T and S are independent.

Alonso and Molenberghs (2007) suggested a multi-trial framework $R_{h}^{2}$: as shown in Equation (2) to enable transportability of results for the information theory approach.

$$EP\left(Y\right)=\;\frac{1}{\left(2\pi e\right)}e^{2h\left(Y\right)}$$

where

(2) $$\vartheta_{i}>0\;\forall i,\sum_{i=1}^{N_{q}}\vartheta_{i}=1$$

For *N* trials there are $N_{q}$ possible values of $R_{h_{i}}^{2}$, the $R_{h}^{2}$ for the *i^th^* trial since trials can be clustered depending, say, on q different characteristics (e.g. centre, country, treating physician). There are many different choices for the set of unknown weights, $\vartheta_{i}$ in (2). The choice of which leads to an uncountable set of parameters, $\Omega_{h}$, each parameter of which could act as a single meaningful measure of $R_{h}^{2}$ in the multi-trial setting:

(3) $$\Omega_{h}=\left\{\Phi_{h}:\Phi_{h}=1-\overset{N_{q}}{\underset{i=1}{\sum }}\vartheta_{i}e_{i}^{-2I_{i}\left(S_{i},T_{i}\right)},where\;\vartheta_{i}>0\;\forall i,\sum_{i=1}^{N_{q}}\vartheta_{i}=1\right\}$$

where $\Phi_{h}$ are the parameters of the set $\Omega_{h}$. Alonso and Molenberghs (2007) highlighted the likelihood reduction factor (LRF) as a good candidate from $\Omega_{h}$ which provides a useful route to defining $\sum_{i=1}^{N_{q}}\vartheta_{i}$. The LRF is a measure of information gain that has been considered under an information theory framework by several authors (Brillinger 2004; Joe 1989; Kullback 1997; Linfoot 1957).

The LRF is particularly useful for surrogacy evaluation as it ranges in the unit interval and has a consistent interpretation across settings: this is a key point as previous approaches could not provide this. Furthermore, it is possible that a high-dimension integral would be needed in the calculation of *I(T,S)* which the LRF avoids, and as we will expand on in section 2.1.1.1 the LRF provides consistent estimation of $R_{h}^{2}$ (Alonso et al. 2016; Alonso and Molenberghs 2007). Finally, previous approaches to surrogacy assessment relied on computationally intensive joint models of S and T, but the LRF assesses just the conditional model of T|S and the marginal model of T and hence avoids this issue.

##### 2.1.1.1. LRF at the individual level: the continuous setting

The LRF was proposed by Alonso et al. (2006) based on the ideas of Kent (1983). At the individual level, the LRF is based on the amount of information gained about the true outcome after accounting for the surrogate which was proposed as a general measure of correlation. Alonso et al. (2005) proposed modelling (4) and (5) for each trial *i* (linear models are presented here, whereas generalised linear models were originally given):

(4) $$T_{ij}=\mu_{i}+\beta_{i}Z_{ij}+\;\varepsilon_{T_{ij}}$$

(5) $$T_{i}=\theta_{0_{i}}+\theta_{1_{i}}Z_{ij}+\theta_{2_{i}}S_{ij}+\varepsilon_{T|S_{ij}}$$

where: $\theta_{0_{i}}$ and $\mu_{i}$ are intercept parameters with and without adjustment for the surrogate; $\beta_{i}$ is the treatment effect parameter for the true outcome; $\theta_{1_{i}}\;and\;\theta_{2_{i}}$ are treatment and surrogate parameters for the model with adjustment for the surrogate. The amount of information on the true outcome gained from the surrogate is calculated via the difference in the log-likelihood between (4) and (5) which is formally expressed as $G_{i}^{2}$, for each trial *i*. $LL_{0}$ is the log-likelihood for the unsaturated model, in this case (4), and $LL_{1}$ for the saturated model, (5), for trial *i*. $G_{i}^{2}=2\left(LL_{1}-LL_{0}\right)$.

The LRF is then calculated:

(6) $$LRF=1-\frac{1}{N}\sum_{i=1}^{N}\exp\left(-\frac{G_{i}^{2}}{n_{i}}\right)$$

To demonstrate the link between the LRF and $R_{h}^{2}$, consider the *i^th^* trial and joint density function $f(T_{i},S_{i}|\theta_{i})$ of $\left(T_{i},S_{i}\right)$, where we have $\theta_{i}=\left(\theta_{{*}_{i}},\theta_{2_{i}}\right)$. Where $\theta_{2_{i}}$ represents the dependence between S and T, ${{\hat{\theta }}_{*}}_{i}$ is the maximum likelihood estimator under the null hypothesis of independence ($\theta_{2_{i}}=0$), and ${\hat{\theta }}_{i}$ is the maximum likelihood estimator for the saturated model. We can express $\frac{1}{n_{i}}G_{i}^{2}=\frac{1}{n_{i}}\sum_{i}[log\{f(t_{i}|s_{i},{\hat{\theta }}_{i})\}-log\left\{f\left(t_{i}|{\hat{\theta_{*}}}_{i}\right)\right\}]$. If ${\hat{\theta_{*}}}_{i}$ converges to $\theta_{{*}_{i}}$ in probability then $\frac{1}{n_{i}}G_{i}^{2}\to I_{i}\left(S_{i},T_{i}\right)$ under general regularity conditions, hence $\frac{1}{n_{i}}G_{i}^{2}$ is a consistent estimator of $I_{i}\left(S_{i},T_{i}\right)$.

Using the estimator $\frac{1}{n_{i}}G_{i}^{2}$ of $I_{i}\left(S_{i},T_{i}\right)$ we have $LRF={\hat{R}}_{h}^{2}=1-\sum_{i=1}^{N_{q}}\frac{1}{N}e^{-\frac{1}{n_{i}}G_{i}^{2}}$, which is a special case of $R_{h}^{2}$ in (3) where $\vartheta_{i}=\frac{1}{N}.$Therefore, the LRF is a consistent estimator of $R_{h}^{2}$ (Alonso and Molenberghs 2007; Brillinger 2004). For a full proof see the supplementary material of (Alonso and Molenberghs 2007).

##### 2.1.1.2. LRF at the individual level: extension to the binary-ordinal setting

The LRF can be used to calculate $R_{h}^{2}$ for a binary surrogate and ordinal true outcome. At the individual level, the LRF can be applied in the binary-ordinal setting in the same manner as in the continuous case using (6), based in this case on the difference $G_{2}=2\left(LL_{1}-LL_{0}\right)$ of the following proportional odds models:

(7) $$logit\left\{P\left(T_{ij}\leq w\right)\right\}=\mu_{T_{w_{i}}}+\beta_{i}Z_{ij}$$

(8) $$logit\left\{P\left(T_{ij}\leq w\right)\right\}=\theta_{0_{w_{i}}}+\theta_{1_{i}}Z_{ij}+\theta_{2_{i}}S_{ij}$$

where w=1,….,W−1, and W is the number of categories in the ordinal true outcome. For trial *i*, $\mu_{T_{w_{i}}}$ and $\theta_{0_{w_{i}}}$ are intercept parameters for each cut point of the ordinal true outcome, $\beta_{i}$ and $\theta_{1_{i}}$ represent the treatment effect on the true outcome and $\theta_{2_{i}}$ is the surrogate parameter. Again, the LRF is based on the amount of information gained on the true outcome after adjusting for the surrogate for each trial.

However, in the case of discrete outcomes and a family of conditional models, the LRF is bounded above by a number strictly less than one (Kent 1983). Alonso and Molenberghs (2007) showed that $R_{h}^{2}\leq 1-$ $e^{-2H\left(T\right)}$, where *H(*T) represents the entropy of T. They also suggested that *H(*T) can be approximated based on the log-likelihood of the intercept-only model of true outcome ($logit\left\{P\left(T_{ij}\leq w\right)\right\}=\theta_{3}$, where $\theta_{3}$ is the intercept parameter). Alonso and Molenberghs (2007) therefore proposed rescaling $R_{h}^{2}$ as calculated by the LRF in (6) by:

(9) $$\hat{R_{h}^{2}}=\frac{R_{h}^{2}}{1-e^{-2H\left(T\right)}}$$

The LRF thus gives a consistent interpretation at the individual level for both the binary-ordinal and continuous settings.

#### 2.1.2. Trial level: information theory approach

At the trial level, interest is in the relationship between treatment effects on the surrogate and treatment effects on the true outcome. Alonso and Molenberghs (2007) proposed a two-stage approach. At the first stage, the treatment effects for each trial on the surrogate and true outcome are obtained, $\alpha_{i}$ and $\beta_{i}$ respectively. This is done by regressing the surrogate and true outcome on treatment in separate models:

(10) $$S_{ij}=\mu_{S_{i}}+\alpha_{i}Z_{ij}+\varepsilon_{S_{ij}}$$

(11) $$T_{ij}=\mu_{T_{i}}+\beta_{i}Z_{ij}+\varepsilon_{T_{ij}}$$

where $\mu_{S_{i}},\mu_{T_{i}},$ represent the mean intercept and $\alpha_{i},\beta_{i}$ the treatment effects for S and T, respectively.

Using the treatment effect estimates for S and T from these models, ${\hat{\alpha }}_{i}$ and ${\hat{\beta }}_{i}$ respectively, we calculate the information theory surrogacy measure $R_{ht}^{2},$ where the subscript *t* indicates that we are now considering trial-level surrogacy, through:

(12) $$R_{ht}^{2}=\;\frac{EP\left(\hat{\beta }\right)-EP\left(\hat{\beta }\text{|}\hat{\alpha }\right)}{EP\left(\hat{\beta }\right)}$$

where $EP\left(\hat{\beta }\right)$ is the entropy power of the distribution of treatment effect estimates on T across the *i* trials and $EP\left(\hat{\beta }\text{|}\hat{\alpha }\right)$ is the entropy power of the distribution of treatment effect estimates on T given those on S. $R_{ht}^{2}$ can be interpreted as the amount of uncertainty in the treatment effect on T removed through knowledge of the treatment effect on S.

##### 2.1.2.1. The LRF at the trial level: the continuous setting

The LRF can be applied to calculate $R_{ht}^{2}$ in the continuous-continuous case. In order to do this (10) and (11) are again modelled to obtain treatment estimates ${\hat{\alpha }}_{i}$ and ${\hat{\beta }}_{i}$ and ${\hat{\mu }}_{S_{i}}$. At the second stage, two further models of the treatment effect on the true outcome are required:

(13) $${\hat{\beta }}_{i}=\gamma_{3}+\varepsilon_{\beta_{i}}$$

(14) $${\hat{\beta }}_{i}=\gamma_{0}+\gamma_{1}{\hat{\mu }}_{S_{i}}+\gamma_{2}{\hat{\alpha }}_{i}+\varepsilon_{\beta |\mu ,\alpha_{i}}$$

where $\gamma_{3}$ and $\gamma_{0}$ are the intercept parameters with and without adjustment for the surrogate treatment effects and $\gamma_{1}$ and $\gamma_{2}$ are the parameters for the surrogate intercept and treatment effect estimates provided from stage one. The difference in log-likelihood between these two models can then be calculated and the LRF applied as in (15).

(15) $$LRF=\hat{R_{ht}^{2}}=1-\exp\left(-\frac{\;G_{2}}{N}\right)$$

In a similar fashion to the LRF at the individual level, it can be shown that the LRF is a consistent estimator of $R_{ht}^{2}$ (Alonso et al. 2016).

##### 2.1.2.2. The LRF at the trial level: extension to the binary-ordinal setting

In the binary-ordinal setting, the key difference in the approach is in the models used at the first stage. Here a generalised linear and proportional odds model are required for the surrogate and true outcome, respectively:

(16) $$logit\left\{P(S_{ij}=1)\right\}=\mu_{S_{i}}+\alpha_{i}Z_{ij}$$

(17) $$logit\left\{P(T_{ij}\leq w)\right\}=\mu_{T_{w_{i}}}+\beta_{i}Z_{ij}$$

where w=1,….,W−1, and *W* is the number of categories in the ordinal true outcome, $\mu_{T_{w_{i}}}$is the set of intercept parameters for each of the *W-1* cut points of the ordinal true outcome and all other parameters are analogous to the continuous case. The second stage models (13) and (14) can be fitted in the same manner as in the continuous setting using the parameters of (16) and (17), and the LRF applied as in (15). The LRF has a consistent interpretation at the trial level for the continuous-continuous and binary-ordinal settings, and it can easily be seen how this would be the case for other settings.

### 2.2. Confidence intervals – all settings

A confidence interval based on the non-central $\chi^{2}$ distribution for $R_{ht}^{2}$ may be calculated as per (Kent 1983):

$$\left\{1-exp\left(-\frac{\gamma_{1:\alpha /2}\left(G^{2}\right)}{N}\right),1-\exp\left(\frac{\delta_{1:\alpha /2}\left(G^{2}\right)}{N}\right)\right\}$$

where $\gamma_{1:\alpha }$ and $\delta_{1:\alpha }$ are defined by $\left[\chi_{1}^{2}\left\{\gamma_{1:\alpha }\left(G^{2}\right)\right\}\geq G^{2}\right]=\alpha$and $P\left[\chi_{1}^{2}\left\{\delta_{1:\alpha }\left(G^{2}\right)\right\}\leq G^{2}\right]=\alpha ,\;and\;\chi_{1}^{2}$ represents the non-central chi-squared distribution with 1 degree of freedom. The above is true unless $P\left\{\chi_{1}^{2}\left(0\right)\geq G^{2}\right\}>\alpha$ in which case $\gamma_{1:\alpha }\left(G^{2}\right)=0$.

$R_{h}^{2}$ on the other hand has multiple $G_{i}^{2}$. Previous publications have computed non-parametric bootstrap confidence intervals in this setting and we follow that methodology (Alonso et al. 2006).

## 3. Simulation study

### 3.1. Set-up

The practical worth of the approach is demonstrated via a thorough simulation study using R, based on the approach of (Tilahun et al. 2008). Different scenarios were simulated to see how the $R_{\;}^{2}$ measures perform when different numbers of trials and sizes of trial are available. We reported the median point estimate and median upper and lower confidence limits over 250 simulations for each scenario investigated. We use the methodology of the precursor to the information theory approach, the meta-analytical approach, to set up the simulation as conducted by many previous authors Tilahun et al. (2008). The normal joint mixed model (17) gives the basis for the data generation:

(18) $$S_{ij}=\mu_{S}+m_{S_{i}}+\alpha Z_{ij}+a_{i}Z_{ij}+\varepsilon_{S_{ij}}$$

$$T_{ij}=\mu_{T}+m_{T_{i}}+\beta Z_{ij}+b_{i}Z_{ij}+\varepsilon_{T_{ij}}$$

where $(\mu_{s}$, $\mu_{T}$) and (α, β) are fixed intercepts and treatment effects, respectively. ($m_{S_{i}},m_{T_{i}})$ and $(a_{i},b_{i}$) are random intercepts and treatment effects for the $i^{th}$ trial, respectively. $(\varepsilon_{S_{ij}}$,$\varepsilon_{T_{ij}})$ ~ N(0,∑) and random effects, ${\left(m_{S_{i}},m_{T_{i}},a_{i},b_{i}\right)}^{T}$ ~ $N\left(0,D\right)$, where:

$$D=3\left(\begin{array}{cccc} 1 & 075 & 0 & 0\\ 0.75 & 1 & 0 & 0\\ 0 & 0 & 1 & \rho \\ 0 & 0 & \rho & 1 \end{array}\right),where\;R_{ht}^{2}=\rho^{2},$$

$$\sum =3\left(\begin{array}{cc} 1 & \psi \\ \psi & 1 \end{array}\right),where\;R_{h}^{2}=\psi^{2}.$$

Specific values of *D* and ∑ were chosen in line with Tilahun et al. (2008) as were individual trial intercept and treatment parameters for S and T which were set to $\mu_{s}$= 0.50, $\mu_{T}$= 0.45, α = 0.05, and β = 0.03. Their values do not influence the true strength of surrogacy.

Four surrogacy scenarios were simulated: strong, with $R_{ht}^{2}=\rho^{2}=0.90$ and $R_{h}^{2}=\psi^{2}=0.64$; weak, with $R_{ht}^{2}=\rho^{2}=0.30$ and $R_{h}^{2}=\psi^{2}=0.30$; or to have discordant levels of surrogacy at trial and individual level, $R_{ht}^{2}=\rho^{2}=0.90$ and $R_{h}^{2}=\psi^{2}=0.30$; or $R_{ht}^{2}=\rho^{2}=0.30$ and $R_{h}^{2}=\psi^{2}=0.64$. After simulating a continuous S and T these were then dichotomised or categorised to represent a binary S and ordinal T. T was set to have seven categories and its distribution was simulated to follow what might be observed in the Oxford Handicap Scale (Van Swieten et al. 1988) investigated in the stroke case study (section 4). We also investigate the setting where the ordinal outcome does not fulfil the proportional odds assumption, by changing for one treatment arm one of the quantiles at which the continuous T is cut to generate the ordinal categorical T. Trial sizes were set to 60, 100, and 300 patients. There were 5, 10, 20 or 30 trials in each simulated data set. There were 250 datasets simulated for each scenario: a total of 15,000 simulations covering all combinations of the strength of surrogacy (4), trial size (3) and number of trials (4) scenarios and in addition the non-proportional odds setting with strong surrogacy for all trial size and number of trials scenarios.

At the individual level in the discrete binary-ordinal case, information theory explores surrogacy at the observed rather than latent scale, and therefore the strength of surrogacy is expected to be lower than on the latent continuous level (Tilahun et al. 2008). This reflects reality, since for example binary measures often represent latent continuous variables and a binary surrogate would be expected to provide less information than a continuous one. Therefore, we expect the maximum surrogacy strength achievable in the observed binary-ordinal setting to be much lower than the ‘true’ strength of surrogacy set at the latent level. We investigated the individual level surrogacy ceiling for a binary surrogate with an ordinal true outcome by further investigating the ideal scenario where $R_{ht}^{2}=\rho^{2}=0.90$ and $R_{h}^{2}=\psi^{2}=1$. In this case, 250 data sets were simulated for each scenario: a total of 3,000 simulations covering all combinations of the trial size (3) and number of trials (4) scenarios.

### 3.2. Results

For strong surrogacy $\hat{R_{h}^{2}\;}$ converges to around 0.30 (Table 1) for larger numbers of trials and trial sizes; this is much lower than the 0.64 strength simulated on the latent continuous scale. Equally, for weak surrogacy $\hat{R_{h}^{2}\;}$ converges to around 0.13 (Table 2) which is again much lower than the strength of 0.30 simulated on the latent scale. Simulations for the ‘perfect’ surrogate with $R_{h}^{2}$ = 1 converge to around $\hat{R_{h}^{2}\;}$ = 0.48, the ceiling for this binary surrogate for an ordinal true outcome generated from a latent continuous measure, see Table 3.

**Table 1.** Simulation study results: Strong surrogacy. True values on the latent continuous scale used to generate data are trial-level surrogacy $R_{ht}^{2}$ = 0.90, and individual-level surrogacy $R_{h}^{2}$ = 0.64 (at the individual level we expect strength of surrogacy in the binary-ordinal setting to be low due to loss of information from moving from continuous to categorical outcomes). 250 simulations were performed for each of the scenarios reported in the table. We present the number and size of trials simulated; the median $R_{2}$of the 250 simulations; median lower and upper limits of the 95% confidence intervals.

		$R_{ht}^{2}$: Trial-level surrogacy			$R_{h}^{2}:$Individual-level surrogacy		
Numberof trials	Trial size	Median $\hat{R_{ht}^{2}}$	Lower 95%CI	Upper 95%CI	Median $\hat{R_{h}^{2}}$	Lower 95%CI	Upper 95%CI
5	60	0.930	0.404	0.998	0.308	0.228	0.398
5	100	0.934	0.429	0.998	0.307	0.242	0.371
5	300	0.948	0.501	0.999	0.305	0.267	0.343
10	60	0.833	0.349	0.982	0.304	0.245	0.367
10	100	0.847	0.411	0.983	0.298	0.252	0.344
10	300	0.895	0.541	0.989	0.299	0.271	0.325
20	60	0.793	0.454	0.952	0.297	0.257	0.341
20	100	0.826	0.522	0.960	0.300	0.268	0.334
20	300	0.871	0.622	0.970	0.293	0.274	0.312
30	60	0.783	0.512	0.929	0.297	0.263	0.332
30	100	0.823	0.588	0.944	0.296	0.270	0.323
30	300	0.866	0.668	0.958	0.293	0.278	0.310

**Table 2.** Simulation study results: weak surrogacy. True values on the latent continuous scale used to generate data are trial-level surrogacy $R_{ht}^{2}$ = 0.30, and individual-level surrogacy $R_{h}^{2}$ = 0.30 (at the individual level we expect strength of surrogacy in the binary-ordinal setting to be low due to loss of information from moving from continuous to categorical outcomes). 250 simulations were performed for each of the scenarios reported in the table. We present the number and size of trials simulated; the median $R_{\;}^{2}$ of the 250 simulations; median lower and upper limits of the 95% confidence intervals for the 250 simulations.

		$R_{ht}^{2}$: Trial-level surrogacy			$R_{h}^{2}:$Individual-level surrogacy		
Numberof trials	Trial size	Median $\hat{R_{ht}^{2}}$	Lower 95%CI	Upper 95%CI	Median $\hat{R_{h}^{2}}$	Lower 95%CI	Upper 95%CI
5	60	0.643	0.028	0.974	0.143	0.086	0.231
5	100	0.682	0.039	0.979	0.140	0.092	0.204
5	300	0.670	0.038	0.977	0.135	0.107	0.171
10	60	0.429	0.012	0.866	0.144	0.104	0.206
10	100	0.393	0.009	0.843	0.137	0.105	0.183
10	300	0.385	0.009	0.832	0.134	0.113	0.158
20	60	0.265	0.010	0.656	0.138	0.114	0.183
20	100	0.303	0.021	0.676	0.136	0.115	0.170
20	300	0.311	0.027	0.673	0.131	0.118	0.149
30	60	0.243	0.019	0.568	0.141	0.122	0.179
30	100	0.271	0.033	0.589	0.136	0.119	0.164
30	300	0.304	0.054	0.610	0.132	0.121	0.147

**Table 3.** Simulation study results: Ceiling effect. True values on the latent continuous scale used to generate data are trial-level surrogacy $R_{ht}^{2}$ = 0.90, and individual-level surrogacy $R_{h}^{2}$ = 1 (at the individual level we expect strength of surrogacy in the binary-ordinal setting to be low due to loss of information from moving from continuous to categorical outcomes). 250 simulations were performed for each of the scenarios reported in the table. We present the number and size of trials simulated; the median $R_{2}$ of the 250 simulations; median lower and upper limits of the 95% confidence intervals for the 250 simulations.

		Individual-level surrogacy		
Numberof trials	Trial size	Median $\hat{R_{h}^{2}}$	Lower 95%CI	Upper 95%CI
5	60	0.539	0.398	0.599
5	100	0.548	0.429	0.598
5	300	0.516	0.444	0.569
10	60	0.514	0.419	0.562
10	100	0.510	0.426	0.556
10	300	0.484	0.424	0.540
20	60	0.492	0.425	0.527
20	100	0.500	0.441	0.535
20	300	0.489	0.438	0.520
30	60	0.494	0.438	0.521
30	100	0.488	0.439	0.517
30	300	0.478	0.438	0.508

Unlike individual-level surrogacy, trial-level surrogacy, R^2^_ht_, ought to report the same surrogacy strength at the latent and explicit scales (Tilahun et al. 2008). However, there appears to be some underestimation of $\hat{R_{ht}^{2}}$ for strong surrogacy even where trial sizes are large (Table 1). This is in line with results in the continuous-binary and binary-binary settings (Pryseley et al. 2007; Tilahun et al. 2008). Conversely, where surrogacy is set to be weak (Table 2) there is overestimation of $R_{ht}^{2}$ for small trial sizes. Further examination showed this was due to overfitting to the resultant small number of data points (one for each trial) in the regression model used at the second stage of $R_{ht}^{2}$ modelling.

$\hat{R_{ht}^{2}}$ and $\hat{R_{h}^{2}}$ estimates where surrogacy strengths differ at trial and individual levels are similar to where surrogacy strengths are consistent, see Table 4. Deviation from the proportional odds assumption also seems to have little impact on results at either level, see Table 5.

**Table 4.** Simulation study results: differing strengths of surrogacy against the case where surrogacy is strong at both levels. 250 simulations were performed for each of the scenarios reported in the table. We present the number and size of trials simulated; and the median $R_{\;}^{2}$ of the 250 simulations. **^¥^** Both comparisons are between strong level surrogacy at both levels, $R_{ht}^{2}=0.90$ and $R_{h}^{2}$ = 0.64, against the case where surrogacy is strong at the level under consideration but weak (either $R_{ht}^{2}=0.30$ or $R_{h}^{2}$ = 0.30) at the unreported level. The converse case gives comparable results (results not shown).

		Median $\hat{R_{ht}^{2}}$^¥^ Trial-level surrogacy		Median $\hat{R_{h}^{2}}$ ^¥^ Individual-level surrogacy	
Numberof trials	Trial size	$R_{ht}^{2}=0.90$ $R_{h}^{2}$ = 0.64	$R_{ht}^{2}=0.90$ $R_{h}^{2}$ = 0.30	$R_{ht}^{2}=0.90$ $R_{h}^{2}$ = 0.64	$R_{ht}^{2}=0.30$ $R_{h}^{2}$ = 0.64
5	60	0.930	0.905	0.308	0.303
5	100	0.934	0.934	0.307	0.308
5	300	0.948	0.951	0.305	0.302
10	60	0.833	0.823	0.304	0.294
10	100	0.847	0.851	0.298	0.293
10	300	0.895	0.895	0.299	0.291
20	60	0.793	0.750	0.297	0.292
20	100	0.826	0.811	0.3	0.290
20	300	0.871	0.865	0.293	0.287
30	60	0.783	0.734	0.297	0.291
30	100	0.823	0.803	0.296	0.291
30	300	0.866	0.861	0.293	0.288

**Table 5.** Simulation study results: considering proportional versus non-proportional odds. True values on the latent continuous scale used to generate data are trial-level surrogacy $R_{ht}^{2}$ = 0.90, and individual-level surrogacy $R_{h}^{2}$ = 0.64. 250 simulations were performed for each of the scenarios reported in the table. We present the number and size of trials simulated; and the median $R_{2}$ of the 250 simulations.

		Median $\hat{R_{ht}^{2}}$ Trial-level surrogacy		Median $\hat{R_{h}^{2}}$ Individual-level surrogacy	
Numberof trials	Trial size	Proportional	Non- Proportional	Proportional	Non- Proportional
5	60	0.930	0.925	0.308	0.305
5	100	0.934	0.928	0.307	0.308
5	300	0.948	0.947	0.305	0.304
10	60	0.833	0.823	0.304	0.301
10	100	0.847	0.845	0.298	0.292
10	300	0.895	0.890	0.299	0.295
20	60	0.793	0.788	0.297	0.295
20	100	0.826	0.818	0.3	0.295
20	300	0.871	0.866	0.293	0.291
30	60	0.783	0.772	0.297	0.294
30	100	0.823	0.818	0.296	0.293
30	300	0.866	0.862	0.293	0.290

## 4. Case study – CLOTS3

The case study, conducted using data from the randomised trial Clots in Legs Or sTockings after Stroke (CLOTS) 3 trial (Dennis et al. 2015), aimed to determine whether measures taken within 30 days of a stroke could be used as a surrogate in place of death and disability measured 6 months post stroke.

Venous thromboembolism encompasses the ailments: deep vein thrombosis (DVT), a blood clot in the deep veins of the legs; and pulmonary embolism (PE), where clots detach from the veins and cause blockages to the lungs. Venous thromboembolism can be serious enough to cause death or be so debilitating it hinders rehabilitation. Dennis et al. (2013) showed that 20–42% of stroke patients suffer a venous thromboembolism. This result reflects the fact that stroke patients are typically bedbound and often unable to move one side of their body.

A primary measure of ongoing health and survival measured in patients 6 months post stroke is the Oxford Handicap Scale (OHS) (Van Swieten et al. 1988). This is an ordinal measure on a seven-point scale, ranging from no symptoms up to severe disability and death.

CLOTS3 was a 94 centre randomised clinical trial with 2,876 patients. It was conducted to investigate whether intermittent pneumatic compression (IPC) applied to the legs of acute stroke patients reduced the occurrence of DVT (Dennis et al. 2015). CLOTS3 () showed that IPC reduced the odds of DVT by 30 days [OR 0.65 (95% CI 0.51–0.84; *p* = .001) after adjustment for baseline variables] and had a positive impact on survival at 6 months, HR 0.86 (0.74–0.99), *p* = .042.

**Figure 1.**
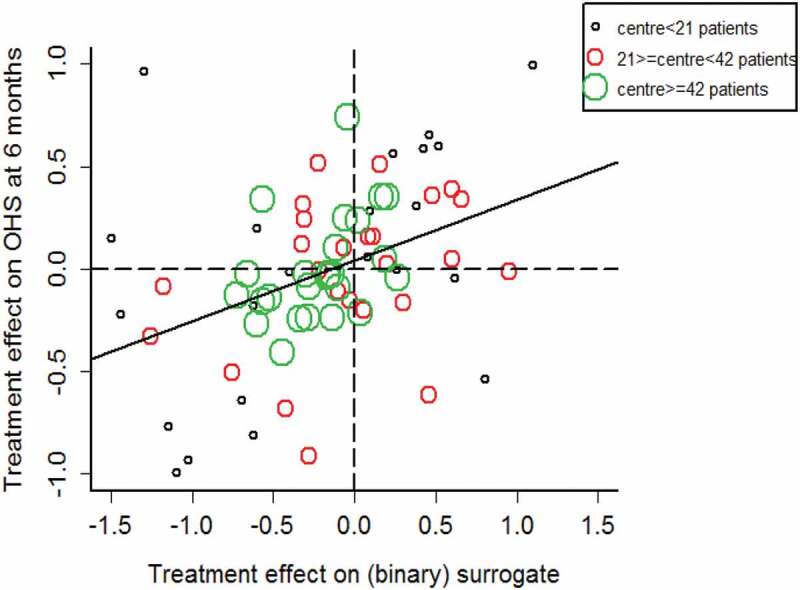
CLOTS3 case study results: Graphical display of information theory surrogacy estimates for binary DVT surrogate and ordinal OHS true outcome; study centre size categorised by the terciles of centre size. The regression line represents the regression of the treatment effects on the true outcome on those for the surrogate. Analysed using a modified information theory approach incorporating a penalized likelihood approach (Firth 1993) to deal with the issue of sparse data.

We used this data set to assess whether the occurrence of DVT, PE or death within 30 days is a surrogate for OHS at 6 months. The information theory approach was applied to investigate this and we used study centres in place of trials (Abrahantes et al. 2004). The results shown in Table 6 and Figure 1 indicate that DVT is not a good surrogate for OHS, as $\hat{R_{h}^{2}}$ is 0.173 95% CI (0.141, 0.188) and $\hat{R_{ht}^{2}}$ is 0.186 95% CI (0.048, 0.374). While there is no established cut-off corresponding to a ‘valid’ surrogate previous publications have suggested that surrogates that exceed 0.80 at both levels can be deemed valid, while if surrogacy strength at either level is below 0.50 surrogacy strength is poor (Alonso et al. 2016). Therefore, these results suggest a poor surrogate.

**Table 6.** CLOTS3 case study results: Information theory surrogacy estimates for binary DVT surrogate and ordinal OHS true outcome; analysed using a modified information theory approach incorporating a penalized likelihood approach (Firth 1993) to deal with the issue of sparse data.

$R_{h}^{2}$ Individual level	$R_{ht}^{2}$ Trial level
0.173	0.186
95% CI (0.141, 0.188))	95% CI (0.048,0.374)

A sensitivity analysis was conducted to assess whether the potential bias witnessed in the simulation study (where underestimation increased with increased number of trials) may have influenced our results. We regrouped centres so there were fewer groups (results not shown), and we found that while some level of underestimation had taken place the point estimates were still comfortably under 0.50 and therefore our conclusions did not change. Further information on the case study is provided in Appendix A.

## 5. Discussion

The information theory approach of Alonso and Molenberghs (2007) has previously been extended to failure time outcomes (Pryseley et al. 2011); repeated measures (Alonso et al. 2006); a continuous surrogate and binary true outcome (Pryseley et al. 2007); and binary outcomes (Tilahun et al. 2008). This paper complements these by extending the methodology to the case of a binary surrogate and an ordinal true outcome.

A major strength of the work presented is the wide range of scenarios considered in the simulation study that evaluated the performance of the extended methodology measures of individual-level surrogacy, $R_{h}^{2}$, and trial-level surrogacy, $R_{ht}^{2}$ in the binary-ordinal context. Extending previous simulation studies in this area (Tilahun et al. 2007, 2008) we assessed weak strengths of surrogacy, discordant levels of surrogacy at trial and individual levels and investigated the ceiling effect present when using binary and ordinal outcomes. We also completed the first assessment via simulation of the non-proportional odds scenario for ordinal outcomes. A further benefit was the opportunity to provide a clear answer to a question of clinical interest regarding deep vein thrombosis, DVT, as a potential surrogate for long-term outcome following stroke the Oxford Handicap Scale, OHS, using data from the CLOTS (Dennis et al. 2015) randomised controlled trial.

As might have been expected, the simulation study showed that a binary surrogate is less informative than its latent counterpart at the individual level; the ceiling for the binary-ordinal setting is around half the strength of that simulated on the underlying continuum.

Some unexpected underestimation of R^2^_ht_ was observed; we speculate that this is due to inefficiencies in estimation through a combination of the use of a two-stage estimation approach and the involvement of discrete outcomes. Furthermore, overestimation of R^2^_ht_ occurred for weak surrogacy and small numbers of trials, due to overfitting at the second stage of modelling. Assessments of surrogates of this kind might lead researchers to believe incorrectly that they are valid. This is likely to be an issue regardless of the setting (binary-continuous, continuous-continuous etc.) and has not previously been identified. These two sources of bias at the trial level, overfitting and inefficiency, point to some practical issues with the two-stage modelling approach and require further investigation. Deviations from the proportional odds assumption or discordant surrogacy strength at trial and individual levels had little impact on $\hat{R_{ht}^{2}}$ or $\hat{R_{h}^{2}}$ results with positive implications for the robustness of this surrogacy assessment approach.

In future work, it would be interesting to study the underestimation found in this work in more detail – perhaps in the context of contrasting settings, e.g. time-to-event or repeated measures. Nevertheless, if inefficiency is the root cause of underestimation the discrete case is likely to be the most severely affected. In the discrete outcome setting the issues of estimation in the presence of separation (perfect agreement between two discrete outcomes) is one that might have a large impact on results. Our simulations did not consider small trial sizes where separation is likely to be a substantial issue; however, this important topic should be considered in more detail alongside potential solutions. Equally, it would be worth establishing if the overfitting witnessed in the case of weak surrogacy is systemic to all settings of the information theory approach.

Overall results from the simulation show that the information theory approach works well in general in the binary-ordinal context, although some issues concerning the two stage nature of the modelling approach for $R_{ht}^{2}$ have been identified. Methodologically we have seen that across settings the information theory approach is readily applied and provides a consistent interpretation. The methodological extensions reported here will enable researchers working in clinical areas where ordinal outcomes are important to investigate surrogacy. This work provides further confirmation that information theory is a practical and methodologically sound approach to surrogacy evaluation.
