# Supplementary material for: Evaluation of surrogacy in the multi-trial setting based on information theory: an extension to ordinal outcomes
Source: J Biopharm Stat. 2019 Dec 30;30(2):364–76. doi: 10.1080/10543406.2019.1696357 (PMC7048082; doi:10.1080/10543406.2019.1696357)
Supplement: Supplemental Material [file LBPS_A_1696357_SM9798.docx]

Appendix A: Clots3 case study


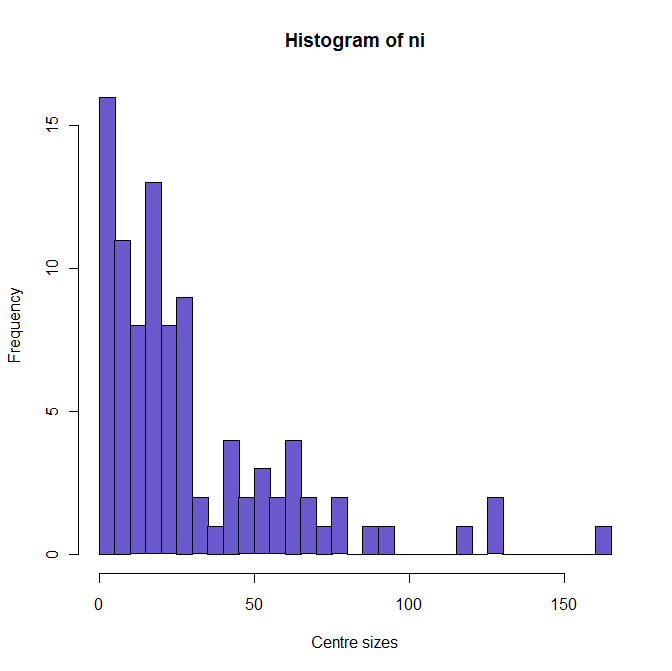


Figure A1: *Histogram of centre sizes*

For an introduction to the CLOTS3 trial see Section 4 or Dennis, Sandercock et al. (2015). Additional information required to understand the surrogacy assessment conducted in Section 4 is outlined below.

General information

CLOTS3 had 35 patients who had missing information for the true, surrogate or treatment outcome, these patients were removed from the analysis leaving 2841 patients. There were 94 centres ranging in size from 1-161 patients, see Figure A1. There were 26 centres that had fewer than ten patients. Twelve of these had fewer than three patients. Models based on centres of very small sizes failed. Since this was the case, the 26 centres with fewer than ten patients were grouped. They were grouped into four groups of size: 27, 30, 31 and 32. Therefore, 72 reformatted groups were used in analysis. The median centre size without consideration of the centres with very small centre sizes was 27. The median size after grouping remained 27.

Dennis, M., P. Sandercock, C. Graham, J. Forbes, J. Smith and C. T. Collaboration (2015). "The Clots in Legs Or sTockings after Stroke (CLOTS) 3 trial: a randomised controlled trial to determine whether or not intermittent pneumatic compression reduces the risk of post-stroke deep vein thrombosis and to estimate its cost-effectiveness." Health technology assessment (Winchester, England) **19**(76): 1.

Surrogacy assessment using R

We refer to centre level surrogacy rather than trial level surrogacy in what follows.

Both centre and individual level surrogacy assessment rests on parameter estimation, especially in regard to the log likelihood (for each centre in the case of individual level surrogacy and at the second stage of modelling for centre level surrogacy). Centre level surrogacy also relies on estimating parameters of treatment effect on S and T. Below we give the results of the second stage of modelling of trial level surrogacy after treatment effect estimates have been calculated, and for individual level surrogacy once the G_i_^2^ estimates (see section 2.1.1.1) have been calculated for each centre. These analyses have been conducted in RStudio Version 1.0.143.

# Output of calculations from R: individual level surrogacy

> ############################################################################## R2h individual level surrogacy

>

>

> # Code for R2h individual level surrogacy after loop which calculates g2 for each centre

>

> # centrenumber gives the number of centres

> centrenumber

[1] 72

>

> # The simulations showed modelling errors for particular centres to be

extremely rare, however if this does occur the model modRmax below should

be restricted to those that do not fail and the LRF and R2hmax should be

adjusted to reflect the number of centres that ultimately contributed)

>

>

> # g2=2(LL1-LL0) as defined in the paper section 2.1.1.1, ni is the

number of patients per centre

>

> indiv_dat<-cbind(g2,ni)

> indiv_dat

g2 ni

[1,] 1.73825097 31

[2,] 1.69732124 30

[3,] 5.23659815 31

[4,] 9.51199952 27

[5,] 1.65139879 15

[6,] 6.57385316 13

[7,] 2.26641981 15

[8,] 0.97728784 10

[9,] 3.34396128 11

[10,] 2.31123897 11

[11,] 0.15347689 12

[12,] 12.90556471 13

[13,] 7.90286355 14

[14,] 3.53333942 16

[15,] 4.71189294 16

[16,] 2.98041074 17

[17,] 0.24145133 17

[18,] 0.04839433 18

[19,] 3.09715605 17

[20,] 5.54222893 18

[21,] 9.43770805 18

[22,] 1.03121255 19

[23,] 5.67718308 18

[24,] 1.28358940 20

[25,] 1.02395319 20

[26,] 4.43350708 20

[27,] 0.56890576 21

[28,] 2.57643352 21

[29,] 4.20002884 21

[30,] 0.22152395 21

[31,] 8.54549710 21

[32,] 3.11018125 23

[33,] 1.23469565 23

[34,] 4.53325191 24

[35,] 10.05423598 25

[36,] 3.80701304 26

[37,] 0.82755484 26

[38,] 3.42538876 27

[39,] 1.60969222 28

[40,] 16.60900161 28

[41,] 1.83168881 29

[42,] 4.65772516 30

[43,] 3.04250827 30

[44,] 10.45459004 33

[45,] 15.16611112 35

[46,] 5.86987488 39

[47,] 7.09689263 41

[48,] 9.27079753 42

[49,] 3.41664337 43

[50,] 7.91852883 43

[51,] 4.46573007 47

[52,] 6.20777373 47

[53,] 4.53396802 50

[54,] 11.22252828 49

[55,] 0.49203574 54

[56,] 21.37489279 52

[57,] 8.81233887 61

[58,] 35.47392523 59

[59,] 3.25549996 62

[60,] 33.28429349 58

[61,] 6.10435551 64

[62,] 2.60391118 65

[63,] 0.36195419 65

[64,] 12.12359606 75

[65,] 16.33296550 78

[66,] 16.37850091 161

[67,] 7.75438708 126

[68,] 11.54980598 80

[69,] 20.74547912 91

[70,] 30.40071338 117

[71,] 17.42736884 87

[72,] 10.66507755 126

>

> expg2<-sum(exp(-indiv_dat[,1]/indiv_dat[,2]))

> expg2

[1] 59.88135

>

# calculating the likelihood reduction factor (lrf)

> lrf<-1-(expg2/centrenumber)

> lrf

[1] 0.1683146

>

> # calculating the intercept only model to adjust for the fact that r2h

is bounded above by a number strictly less than one in the ordinal-binary setting, see section 2.1.1.2

>

> modRmax<-lrm(tru~1)

>

> # rescaled R2h called R2hmax

> r2hmax<-lrf/(1-exp(2*as.numeric(logLik(modRmax))/sum(ni)))

> r2hmax

[1] 0.1731072

# Output of calculations from R: centre level surrogacy

Please note that at the centre level we are concerned with the predictive ability of the treatment effect estimates of the surrogate on those for the true outcome; statistical significance, while interesting, is not sufficient indication of surrogacy potential.

Given the number of small numbers of patients per centre, parameters were estimated using a modified information theory approach incorporating a penalized likelihood method (Firth 1993) to deal with the issue of sparse data.

Firth, D. (1993). "Bias reduction of maximum likelihood estimates." Biometrika **80**(1): 27-38.

> ######################################################################## Final R output from the second stage of modelling R2ht at the centre level

>

> # Parameter coefficients taken from first stage proportional odds

(bi – treatment effect on T) and logistic regression models (intercept and

treatment effect on S - mui and ai respectively) and ni - the size of

individual centres

>

> centre_dat<-cbind(bi.f,ai.f,mui.f,ni)

> centre_dat

bi.f ai.f mui.f ni

[1,] 1.190690e-01 -0.32217851 -0.18864712 31

[2,] -3.296133e-01 -1.25276297 -1.38629436 30

[3,] -1.996679e-01 0.05889152 -0.19242291 31

[4,] -9.119064e-01 -0.27980789 -0.41333929 27

[5,] 5.630716e-01 0.23500181 -1.84443973 15

[6,] -6.411485e-01 -0.69314718 -0.69314718 13

[7,] 9.674048e-01 -1.30134483 -0.89587973 15

[8,] -5.343857e-01 0.80471896 -0.11157178 10

[9,] -8.110254e-01 -0.62638148 -1.31952866 11

[10,] 2.027405e-01 -0.60198640 -0.09116078 11

[11,] 5.874924e-01 0.42364747 -1.52225976 12

[12,] 6.538291e-01 0.45814537 -0.45814537 13

[13,] -2.232417e-05 0.25541281 -1.35402510 14

[14,] 1.517908e-01 -1.49786614 -0.58157540 16

[15,] 6.181850e-02 0.08017032 -2.11705225 16

[16,] -9.278859e-01 -1.02706154 -1.53788716 17

[17,] -1.783274e-01 -0.62638148 -1.31952866 17

[18,] -4.614218e-02 0.61607184 -0.77022252 18

[19,] -9.929774e-01 -1.09861229 0.40546511 17

[20,] -2.188350e-01 -1.44208997 -1.69340435 18

[21,] 6.001084e-01 0.51480971 -0.73795326 18

[22,] 2.851474e-01 0.09116078 -1.00745151 19

[23,] -7.676442e-01 -1.15129255 -0.92814899 18

[24,] 3.109260e-01 0.37884285 -0.60198640 20

[25,] 9.963969e-01 1.09861330 -1.73460208 20

[26,] -1.574720e-02 -0.40546510 -1.79175946 20

[27,] -1.502229e-02 0.95047905 -2.41681582 21

[28,] 4.886451e-02 0.60198640 -1.18977307 21

[29,] -1.661431e-01 0.30306790 -1.40168019 21

[30,] -8.733643e-02 -1.17568763 0.07707534 21

[31,] -5.078152e-01 -0.75203870 -0.75203870 21

[32,] 5.100611e-01 0.15922687 -1.54552123 23

[33,] -6.149679e-01 0.45814537 -0.92814900 23

[34,] 2.211843e-02 0.20273255 -0.89587973 24

[35,] -1.544658e-01 -0.03226926 -0.94855999 25

[36,] 3.385170e-01 0.66087792 -0.94855999 26

[37,] 2.396523e-01 -0.31207715 0.15792647 26

[38,] 1.574320e-01 0.11157178 -1.49786614 27

[39,] -1.090651e-01 -0.10033535 -0.91126556 28

[40,] -8.378956e-03 -0.21539146 -1.17090290 28

[41,] -6.784252e-01 -0.42109138 -0.75756362 29

[42,] 3.556784e-01 0.48254045 -0.77022252 30

[43,] 1.603271e-01 0.08352704 -1.38281003 30

[44,] 3.139671e-01 -0.31430433 -0.19652129 33

[45,] 1.017703e-01 -0.06806609 -1.23121690 35

[46,] 3.858164e-01 0.60198640 -0.86435067 39

[47,] 5.171184e-01 -0.22184316 -0.54029689 41

[48,] 7.419527e-01 -0.04480608 -0.58380258 42

[49,] 3.538192e-01 0.16271120 -0.53043598 43

[50,] -1.426873e-01 -0.52072693 1.61933922 43

[51,] -1.564419e-01 -0.56748997 0.12565721 47

[52,] -2.575590e-02 -0.65865074 -1.28725940 47

[53,] -2.406051e-01 -0.28768207 -0.60613580 50

[54,] 3.494173e-01 0.20273255 -0.89587973 49

[55,] 3.369968e-01 -0.56423263 -1.17036843 54

[56,] -9.157685e-02 -0.10033535 -0.74696251 52

[57,] -4.095143e-01 -0.44690894 -1.54552123 61

[58,] -2.136143e-01 0.03449644 -0.90996517 59

[59,] 5.013937e-02 0.18597121 -0.42713326 62

[60,] 2.483141e-01 -0.05889152 -1.44518588 58

[61,] -2.366506e-01 -0.13812669 -0.83127387 64

[62,] 2.334911e-01 0.02127981 -0.67186737 65

[63,] -1.308482e-01 -0.72399397 0.23151748 65

[64,] -2.436606e-01 -0.33756434 -1.27187357 75

[65,] -4.611406e-02 0.26727108 -1.00124025 78

[66,] -2.529645e-02 -0.16208014 -1.20939913 161

[67,] -2.703043e-01 -0.60341294 -0.86292413 126

[68,] -2.556107e-02 -0.30512976 -0.59281183 80

[69,] 1.034615e-01 -0.11442079 -1.27187357 91

[70,] -3.769215e-02 -0.16823612 -1.08452685 117

[71,] -8.838037e-02 -0.27749842 -1.22657898 87

[72,] -2.686104e-02 -0.13432648 -0.73650188 126

>

> # cen.f=total number of centres that contribute to the model.

> cen.f

[1] 72

>

> # Unlike the simulation where centre/trial sizes are uniform, centres in the case study are of different sizes. Tibaldi et al. (2003) recommend

weighting by centre size

>

> lrfht1.f<-lm(bi.f~1,weights=ni)

> lrfht2.f<-lm(bi.f~mui.f+ai.f,weights=ni)

>

> summary(lrfht1.f)

Call:

lm(formula = bi.f ~ 1, weights = ni)

Weighted Residuals:

Min 1Q Median 3Q Max

-4.6777 -1.0060 -0.0573 1.5285 4.8841

Coefficients:

Estimate Std. Error t value Pr(>|t|)

(Intercept) -0.01168 0.03838 -0.304 0.762

Residual standard error: 2.046 on 71 degrees of freedom

> summary(lrfht2.f)

Call:

lm(formula = bi.f ~ mui.f + ai.f, weights = ni)

Weighted Residuals:

Min 1Q Median 3Q Max

-4.5264 -1.3873 0.0501 1.1115 5.2065

Coefficients:

Estimate Std. Error t value Pr(>|t|)

(Intercept) 0.053766 0.069978 0.768 0.444911

mui.f 0.008922 0.063654 0.140 0.888944

ai.f 0.324806 0.083122 3.908 0.000215 ***

---

Signif. codes: 0 ‘***’ 0.001 ‘**’ 0.01 ‘*’ 0.05 ‘.’ 0.1 ‘ ’ 1

Residual standard error: 1.872 on 69 degrees of freedom

Multiple R-squared: 0.186, Adjusted R-squared: 0.1625

F-statistic: 7.886 on 2 and 69 DF, p-value: 0.0008236

> logLik(lrfht1.f)

'log Lik.' -29.20858 (df=2)

> logLik(lrfht2.f)

'log Lik.' -21.79803 (df=4)

>

> g2ht.f<-2*(as.numeric(logLik(lrfht2.f))-as.numeric(logLik(lrfht1.f)))

> g2ht.f

[1] 14.82112

>

> r2ht.f<-1-exp(-g2ht.f/cen.f)

> r2ht.f

[1] 0.1860439

>

> # Tibaldi, Fabian, et al. "Simplified hierarchical linear models for the evaluation of surrogate endpoints." Journal of Statistical Computation and Simulation 73.9 (2003): 643-658.
